# Supplementary material for: Mechanisms governing the pioneering and redistribution capabilities of the non-classical pioneer PU.1
Source: Nat Commun. 2020 Jan 21;11:402. doi: 10.1038/s41467-019-13960-2 (PMC6972792; doi:10.1038/s41467-019-13960-2)
Supplement: Supplementary file 7 — Source data [file 41467_2019_13960_MOESM7_ESM.zip › Source_Data/Figure6/Figure6B_MotifScanOutput/FLI1_PU.1/homerResults/motif16.similar.html]

motif16

## Information for motif16

G
C
A
T
C
T
G
A
C
T
G
A
G
T
A
C
G
T
C
A
C
T
A
G
G
A
T
C
  
Reverse Opposite:  

C
T
A
G
A
G
T
C
C
A
G
T
A
C
T
G
G
A
C
T
G
A
C
T
C
G
T
A
  

|  |  |
| --- | --- |
| p-value: | 1e-104 |
| log p-value: | -2.403e+02 |
| Information Content per bp: | 1.611 |
| Number of Target Sequences with motif | 5687.0 |
| Percentage of Target Sequences with motif | 57.16% |
| Number of Background Sequences with motif | 16376.5 |
| Percentage of Background Sequences with motif | 44.88% |
| Average Position of motif in Targets | 98.1 +/- 57.6bp |
| Average Position of motif in Background | 99.9 +/- 67.0bp |
| Strand Bias (log2 ratio + to - strand density) | -0.0 |
| Multiplicity (# of sites on avg that occur together) | 1.39 |
| Motif File: | file (matrix) reverse opposite |

### Similar de novo motifs found

|  |  |  |  |  |  |  |  |
| --- | --- | --- | --- | --- | --- | --- | --- |
| Rank | Match Score | Redundant Motif | P-value | log P-value | % of Targets | % of Background | Motif file |
| 1 | 0.766 | A G T C G C T A C T A G A T G C G C A T A C T G G C T A G A C T C G T A C G T A | 1e-70 | -161.612307 | 25.42% | 17.31% | motif file (matrix) |
| 2 | 0.770 | G A C T C T G A C G T A G A T C T C G A C A T G G A T C G A C T C T A G | 1e-67 | -154.431827 | 27.29% | 19.11% | motif file (matrix) |
